# Case report: XMEN disease: a patient with recurrent Hodgkin lymphoma and immune thrombocytopenia

**DOI:** 10.3389/fmed.2023.1264329

**Published:** 2023-11-29

**Authors:** Pieter F. de Groot, Arjan J. Kwakernaak, Ester M. M. van Leeuwen, Rosalina M. L. van Spaendonk, Evert-Jan Kooi, Daphne de Jong, Taco W. Kuijpers, Josée M. Zijlstra, Godelieve J. de Bree

**Affiliations:** ^1^Division of Clinical Immunology and Allergy, Department of Internal Medicine, Amsterdam University Medical Centers, Amsterdam, Netherlands; ^2^Department of Experimental Immunology, Amsterdam Institute for Infection and Immunity, Amsterdam University Medical Centers, University of Amsterdam, Amsterdam, Netherlands; ^3^Department of Human Genetics, Amsterdam University Medical Centers, Amsterdam, Netherlands; ^4^Department of Pathology, Amsterdam University Medical Centers, Amsterdam, Netherlands; ^5^Department of Paediatric Immunology, Infectious Diseases and Rheumatology, Amsterdam University Medical Centers, Amsterdam, Netherlands; ^6^Division of Haematology, Department of Internal Medicine, Cancer Center Amsterdam, Amsterdam University Medical Centers, Amsterdam, Netherlands; ^7^Division of Infectious Diseases, Department of Internal Medicine, Amsterdam University Medical Centers, Amsterdam, Netherlands

**Keywords:** inborn error of immunity, Classical Hodgkin lymphoma (CHL), immune thrombocytopenia (ITP), hematopoietic stem cell transplantation, XMEN disease

## Abstract

Here we present the case of a 28-year-old man with X-linked immunodeficiency with magnesium defect, Epstein–Barr virus (EBV) infection and neoplasia (XMEN) disease. He presented with immune thrombocytopenia within 1 year after successful autologous hematopoietic stem cell transplantation for recurrent EBV-associated classical Hodgkin lymphoma (CHL). The combination of EBV- associated malignancy, autoimmunity, recurrent airway infections at young age and bronchiectasis, prompted immunological investigation for an inborn error of immunity (IEI). Genetic testing revealed XMEN disease. XMEN disease is characterized by a glycosylation defect due to mutations in the MAGT1 gene. Germline mutations in the MAGT1 gene disrupt glycosylation of the NKG2D receptor in immune cells, including natural killer and CD8-positive T cells, vital for immune surveillance, especially against EBV. Consequently, individuals with XMEN disease, are prone to EBV-associated lymphoproliferative disorders in addition to auto-immunity. Early recognition of adult onset IEI-related B-lymphoproliferative disorders, including CHL is of vital importance for treatment decisions, including (allogeneic) haematopoietic stem cell transplantation and family screening.

## Case description

**FIGURE 1:**
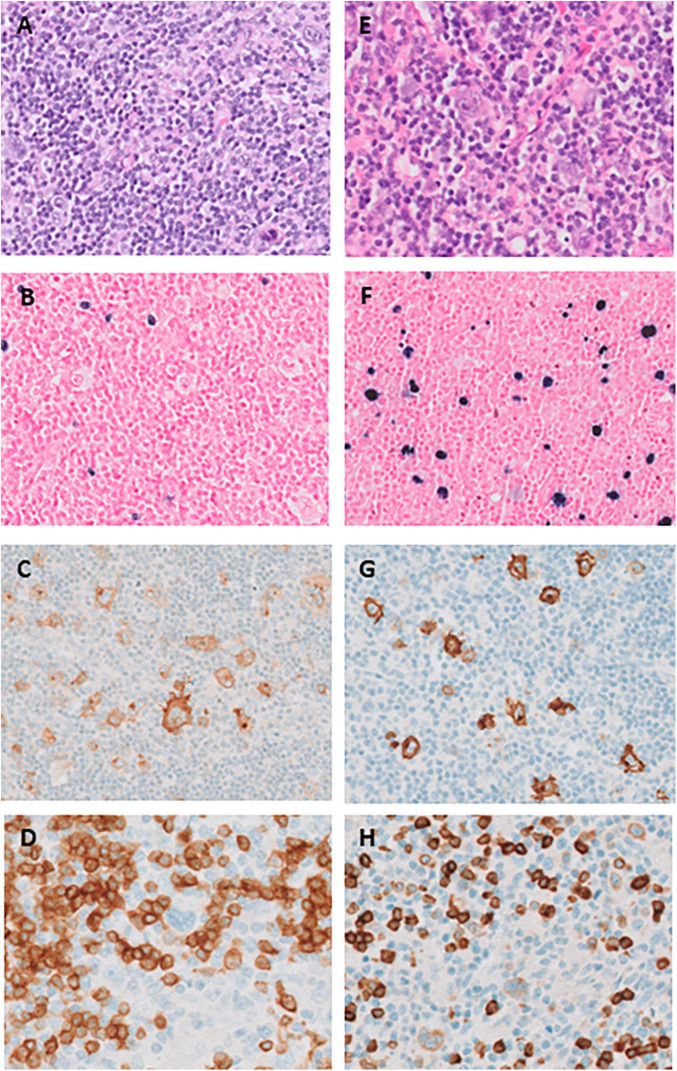
**(A–D)** Histopathology of the supraclavicular lymph node at initial presentation in 2015 (panel **A–D**) and right cervical lymph node at relapse in 2019 (panel **E–H**). **(A)** Hematoxylin-eosin stain shows scattered large Hodgkin Reed-Sternberg (HRS) cells in a reactive background of small lymphocytes, histiocytes and sporadic eosinophilic granulocytes; **(B)** EBER (Epstein–Barr encoding region) shows sparse positive small and slightly enlarged lymphocytes, but HRS-cells are negative; **(C)** CD30 is uniformly positive in HRS-cells as well as in a range of immunoblasts and small lymphocytes; **(D)** CD20 is negative in HRS-cells. **(E)** Hematoxylin-eosin stain shows a larger morphological spectrum of scattered large HRS cells and immunoblasts; **(F)** EBER shows a high proportion of positive cells with a typical range of HRS-cells to small lymphocytes; **(G)**: CD30 is uniformly positive in HRS-cells as well as in a range of immunoblasts and small lymphocytes; **(H)** CD79a positive in these cells.

Our patient was diagnosed in his early twenties with advanced-stage classic Hodgkin lymphoma (CHL) for which he underwent treatment consisting of six courses of escalated BEACOPP (Bleomycin, Etoposide, Adriamycin, Cyclophosphamide, Vincristine, Procarbazine, and Prednisone). At the time of diagnosis, the (18)F-fluorodeoxyglucose positron-emission tomography/computed tomography (FDG-PET-CT) showed pathologic FDG-positive lymphadenopathy both above and below the diaphragm, as well as bronchiectasis. A diagnosis of CHL was confirmed through a supraclavicular lymph node biopsy sample (Figures 1A–D). Four years later, he presented with recurrent CHL, involving cervical, supraclavicular and mediastinal lymph nodes. The diagnosis was confirmed through a cervical lymph node biopsy (Figures 1E–H). He received treatment with three courses of DHAP (Dexamethasone, High dose Cytarabin, Cisplatin), followed by consolidation with BEAM (Carmustine, Etoposide, Cytarabin, and Melphalan) and autologous hematopoietic stem cell transplantation (HSCT). Partial metabolic remission was achieved before HSCT, and complete remission was attained 3 months after HSCT. Six months after the autologous HSCT, the patient developed steroid-refractory immune thrombocytopenia (ITP) responsive to eltrombopag. Consecutive recurrent lymphoma, ITP and in retrospect the presence of bronchiectasis on the initial PET-CT, raised the suspicion of an inborn error of immunity (IEI), leading to his referral for immunological evaluation.

His medical history revealed pneumonia at the age of four and recurrent upper airway infections necessitating antibiotic treatment throughout his primary and high school years. No causative organisms were reported. His parents are in good health, but his mother’s younger brother passed away at the age of two due to acute leukemia of an unknown type. The patient has two healthy sisters. At the time of immunological evaluation, his physical examination yielded unremarkable results. His peripheral blood cell counts were normal. Table 1 provides an overview of laboratory test. Immunological assessment showed panhypogammaglobulinaemia [IgG 4.2 g/L (7–16), IgA 0.13 g/L (0.7–4), IgM 0.71 g/L (0.4–1.3)], low-normal CD4-positive and CD8-positive T cell counts with an inverted CD4/CD8 ratio. We observed a reduction of naïve T-cells and an increase of the fraction of memory and effector-memory T-cell exceeding the normal range for his age and CMV serostatus. His B-cell compartment showed increased numbers, primarily comprised of naïve B-cells. Functional humoral immune reactivity was tested following Pneumovax 23 and tetanus vaccinations, but the patient did not generate specific IgG responses to either vaccine. The patient was IgG seronegative for cytomegalovirus (CMV), herpes simplex virus, and measles, to which he had been vaccinated in the past. Antinuclear antibodies (ANA) were absent. Altogether, this analysis indicated a combined immunodeficiency. Given these immunological deficiencies, along with the presence of bronchiectasis and a history of recurrent upper and lower airway infections, intravenous immunoglobulin (IVIG) therapy was initiated.

**TABLE 1:** Laboratory values at immunological evaluation (18 months after chemotherapy): *percentage of total CD8+ cells, **percentage of total CD4+ cells, ***percentage of total B cells.

Value	Cell counts/percentage (reference range)
Hemoglobulin	**7.9** (8.5–10.5) mmol/L
Platelets	**119** (150–400) (10^9^/L)
Leukocytes	6.1 (4–10.5) (10^9^/L)
Lymphocytes	2.59 (1.0–3.5) (10^9^/L)
CD3+ T cells	0.80 (0.7–2.1) (10^9^/L)
CD4/CD8 ratio	**0.9** (1.0–3.6)
NK cells	0.20 (0.09–0.6) (10^9^/L)
CD8+ T cells	0.36 (0.2–0.9) (10^9^/L)
Naïve CD8+ T cells (RA+/CD27+)	24.4%*
Memory CD8+ T cells (RA-/CD27+)	55.8%*
Effector CD8+ T cells (CD27−)	18.3%* (< 5% in CMV-seronegative)
CD4+ T cells	0.31 (0.3–1.4) (10^9^/L)
Naïve CD4+ T cells (RA+CD27+)	21.6%**
Memory CD4+ T cells (RA-CD27+)	59.3%**
Effector CD4+ T cells (RA-CD27−)	18.7%**
B cells	0.82 (0.1–0.5) (10^9^/L)
Naïve B cells (IgD+/CD27−)	96.9%*** (48.8–76.7)
Non-switched memory B cells (IgD+CD27+)	1.2%*** (8.8–25.6)
Switched memory B cells (IgD-CD27+)	0.1%*** (10.1–25.8)

In bold values outside reference range are depicted.

From whole exome data, we identified genetic variants in the exons and adjacent regions of 431 genes associated with inborn errors of immunity (IEI). We demonstrated a loss-of-function mutation in magnesium transporter 1 (MAGT1), with the patient being hemizygous for the variant NM_001367916.1(MAGT1):c.410delp.(Pro137Glnfs_44). This gene defect aligns with X-linked immunodeficiency with magnesium defect, Epstein–Barr virus (EBV) infection, and neoplasia (XMEN) disease, which falls within the “Immune dysregulation disorders” category according to the classification from the International Union of Immunological Societies Expert Committee. This loss-of-function mutation had not been previously reported. To address the pathogenicity of the hemizygous variant NM_001367916.1(MAGT1):c.410delp.(Pro137Glnfs_44), we followed the guidelines of the American College for Medical Genetics and Genomics (ACMG) (1) for the interpretation of sequence variants. According to this guideline the following considerations were made in the interpretation of the pathogenicity of the variant. According to the algorithm of Abou Tayoun et al. (2), the variant is predicted to result in a loss of function, classified as PVS1 (“pathogenic very strong 1”). PVS1 signifies high confidence in its pathogenic nature. Additionally, loss of function is a known disease mechanism for MAGT1. This variant exists in a biologically significant transcript and is expected to undergo nonsense-mediated decay. Importantly, the variant is not present in the Genome Aggregation Database (gnomAD).^1^ Given that the variant is absent from a large population the variant was considered a moderate piece of evidence for pathogenicity (PM2). When evaluating this genetic variant according to the ACMG/AMP guidelines and considering both PVS1 and PM2 criteria, it falls into Class 4, denoting it as “likely pathogenic.” It is noteworthy that there are no benign frameshift variants in the MAGT1 gene in the control population database.

**FIGURE 2:**
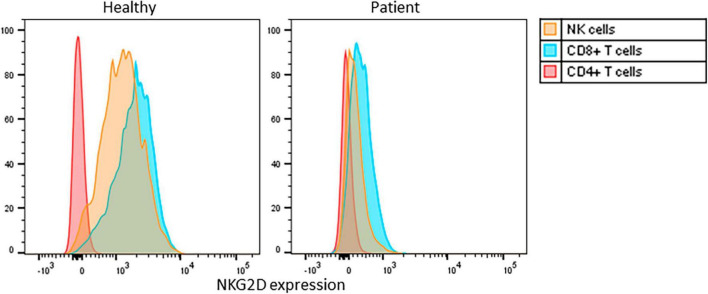
Flowcytometric analysis of cell surface NKG2D expression on PBMC, gated on CD3+CD4+ T cells, CD3+CD8+ T cells, CD3-CD16/56+ NK cells, of a healthy control and the patient. Histograms normalized for cell number per subset. MFI: mean fluorescence intensity for NKG2D cell surface expression on NK-cells (yellow), CD8-positive T-cells (blue) and CD4-positive T-cells (red).

The expression of the cell surface receptor protein NKG2D on NK- and CD8-positive T-cells serves as the gold standard for confirming this diagnosis (3, 4). The patient exhibited markedly decreased NKG2D expression on his CD8-positive T-cells [mean fluorescence index (MFI) 25%] and NK-cells (MFI 8%) compared to a healthy control (MFIs 87 and 84%, respectively) (Figure 2).

XMEN disease is chiefly characterized by immune dysregulation marked by autoimmune conditions (autoimmune hemolytic anemia, ITP), infections (recurrent airway infections), laboratory and functional test aberrations (hypogammaglobulinemia, impaired polysaccharide IgG responses), and EBV-associated lymphoproliferative disorders. Germline mutations in the MAGT1 gene result in a glycosylation defect that principally disrupts proper glycosylation of the NKG2D receptor in immune cells, including NK (natural killer) and CD8-positive T cells, vital for immune surveillance, especially against EBV (3). Thus far, several dozen patients with XMEN have been reported presenting with various types of lymphoproliferative disease (LPD)s that are mostly EBV-associated B-cell lymfoproliferative diseases (B-LPDs). Also, one case of EBV-negative primary cutaneous T-cell lymphoma has been reported (5).

Specifically in XMEN, the deficient MAGT1 protein in NK- and CD8-positive T-cells drives dysregulated EBV control. Therefore, we determined the plasma EBV DNA concentration at several time points and determined the EBV DNA load in lymphocyte cell fractions. The EBV-DNA viral load in plasma was 21,000 IU/ml initially, which spontaneously normalized to < 1,000 IU/ml over the course of several months. The lymphocytes were FACS-sorted into subsets, and EBV was present in B-cells (> 100,000 IU/ml), as well as in T-cells (36,000 IU/ml), monocytes (2,100 IU/ml), and NK cells (< 1,000 IU/ml). It should be noted that an undetectable plasma EBV-load does not preclude the development of EBV-associated LPD (6).

As in our patient, classic Hodgkin lymphoma (CHL), with or without EBV-association is the most commonly reported LPD in XMEN patients (7). At relapse, however, the histology differed in various aspects. Most importantly, a large EBER-positive population was present, covering the complete spectrum from large HRS-(like) cells, intermediate immunoblast-like cells to small lymphocytes. Aberrant CD79A expression was seen in a minority of HRS-(like) cells. While classification as relapsed CHL may be justified in the clinical context, these features should raise suspicion for an underlying IEI. According to the current WHO-HAEM5 unifying nomenclature that recognizes the characteristics of the spectrum of immune dysregulation-associated LPDs, the observed lymphoproliferation should now be classified as “polymorphic B-LPD, CHL-like, EBV-associated, IEI-setting (XMEN)” (8).

In light of a unifying approach to immune dysregulation/deficiency-associated LPDs in WHO-HAEM5, it is likely that a significant proportion of EBV-associated CHL in IEI-settings will now be classified as such in the future, significantly improving the communication between hematopathologists and clinicians to support personalized treatment decisions. In our patient, the recognition of the underlying IEI leads us to consider alloHSCT to prevent subsequent potentially life-threatening EBV-associated lymphoma.

## Treatment

The only curative treatment option for XMEN disease is an allogeneic hematopoietic stem cell transplantation (alloHSCT) to correct the primary immune defect. Generally, alloHSCT for IEI is more frequently performed in infants and children than in adolescents and adults. Current data on alloHSCT for IEI in adults are limited. A recent retrospective study shows that adolescent and adult patients with monogenic severe IEI who underwent alloHSCT had an improved clinical outcome compared to a control cohort, with disease-free survival at 5 years of 58 versus 33% in non-transplanted patients, despite transplant-related mortality of 13% at 1 year (9). For XMEN disease, there are only six published cases on HSCT in adolescents and young adults with variable outcomes (10–12). The studies (10–12), describe *n* = 2, *n* = 3, and *n* = 1 cases of HSCT in XMEN disease, respectively. The study by Li et al. (10) only briefly mentions HSCT in 2 patients without providing a description of outcomes. Two of the described cases by Dimitrova et al. (11) have had a fatal outcome in part due to transplantation-related complications. In Klinken et al. (12) a patient is described with good (short term) outcome of transplantation. Although the number of cases is small, the presence of XMEN-related organ damage seems to be a negative prognostic factor. Notably, severe bleeding complications, out of proportion to the depth of thrombocytopenia, have been reported after alloHSCT in XMEN patients. The underlying cause that triggers bleeding complications remains thus far unclear (10, 11). The medical history of our patient revealed recurrent epistaxis from the left nostril, which resolved after elective chemical cautery. Also, in 2017, he reported hemoptysis, which was confirmed by bronchoscopy and attributed to bronchiectasis and airway infection. His International Society of Thrombosis and Haemostasis Bleeding Assessment Tool score was 3, within the normal range. Clotting times were normal, as were von Willebrand and factor VIII activity. platelet function assay with adenosine diphosphate (PFA ADP) was slightly raised (129 s), but in light of slight thrombocytopenia (117 cells* 10^9/L, normal range 150–400), the test was considered normal.

Finally, in making a decision on alloHSCT in patients with IEI, including XMEN, disease-related risks, including lymphoid malignancies, should be balanced against alloHSCT-related risks and the availability of a suitable donor (13, 14). We discussed the option for alloHSCT with our patient and at the time of drafting the manuscript he is considering this option.

## Conclusion

With the description of this XMEN-associated case, we aim to raise awareness of underlying IEI in young adults presenting with EBV-associated LPDs, including EBV-associated lymphoma, especially when combined with autoimmune disorders, a history of recurrent infections or bronchiectasis. Early recognition is pivotal for preventing subsequent EBV-associated LPDs, other IEI autoimmune and syndromic complications, and end-organ damage. The high incidence of unexplained bleeding complications in XMEN disease requires specific attention. A timely (pre-emptive) allogenic HSCT trajectory may be curative and could be considered.

## Data availability statement

The original contributions presented in this study are included in this article/supplementary material, further inquiries can be directed to the corresponding author.

## Ethics statement

Written informed consent was obtained from the individual(s) for the publication of any potentially identifiable images or data included in this article.

## Author contributions

PG: Conceptualization, Project administration, Writing – original draft, Writing – review and editing. AK: Supervision, Writing – original draft, Writing – review and editing. EL: Formal analysis, Visualization, Writing – review and editing. RS: Writing – review and editing. E-JK: Visualization, Writing – review and editing. DJ: Writing – original draft, Writing – review and editing. TK: Writing – review and editing. JZ: Writing – review and editing. GB: Conceptualization, Funding acquisition, Validation, Writing – original draft, Writing – review and editing.

## Footnotes

1
https://gnomad.broadinstitute.org/
